# Perceptions of Practicing Physicians and Members of the Public on the Attributes of a “Good Doctor”

**DOI:** 10.3390/healthcare10010073

**Published:** 2021-12-31

**Authors:** Keren Dopelt, Yaacov G. Bachner, Jacob Urkin, Zehava Yahav, Nadav Davidovitch, Paul Barach

**Affiliations:** 1 Department of Public Health, Ashkelon Academic College, Ben Tzvi St. 12, Ashkelon 78211, Israel; 2 School of Public Health, Faculty of Health Sciences, Ben-Gurion University of the Negev, P.O. Box 653, Beer-Sheva 84105, Israel; bachner@bgu.ac.il (Y.G.B.); Urkin@bgu.ac.il (J.U.); Zehavayahav@bgu.ac.il (Z.Y.); nadavd@bgu.ac.il (N.D.); 3 Department of Pediatrics, School of Medicine, Jefferson College of Population Health, Wayne State University, Philadelphia, PA 19107, USA; Paul.Barach@jefferson.edu; 4 Interdisciplinary Research Institute for Health Law and Science, Sigmund Freud University, A-1020 Vienna, Austria

**Keywords:** traits of a “good doctor”, technical skills, interpersonal skills, medical education, patient-centered care

## Abstract

Since physician–patient relationships are a central part of the medical practice, it is essential to understand whether physicians and the general public share the same perspective on traits defining a “good doctor”. Our study compared the perceptions of physicians and members of the public on the essential traits of a “good doctor.” We conducted parallel surveys of 1000 practicing specialist-physicians, and 500 members of the public in Israel. Respondents were asked about the two most important attributes of a “good doctor” and whether they thought the physicians’ role was to reduce health disparities. Many physicians (56%) and members of the public (48%) reported that the role of physicians includes helping to reduce health disparities. Physicians emphasized the importance of non-technical skills such as humaneness and concern for patients as important traits of a “good doctor,” while the public emphasized professional and technical skills. Internal medicine physicians were more likely than surgeons to emphasize humaneness, empathy, and professionalism. Future research should focus on actionable approaches to bridge the gap in the perceptions between the groups, and that may support the formation of caring physicians embedded in a complex array of relationships within clinical and community contexts.

## 1. Introduction

*"A good physician treats the disease. The great physician treats the patient who has the disease."*

(Sir Dr. William Osler)

From ancient times, the medical profession has been considered one of the noblest professions. Physicians were honored and perceived as having divine virtues [1]. Their recommendations were accepted as if they were “the word of God” [2]. Physicians seldom explained their medical decisions and certainly did not share decision-making with the patient. Many patients did not expect an equitable relationship and accepted their doctors’ recommendations without question. A 1984 Journal of the American Medical Association article found that 47% of patients prefer their doctor to make decisions regarding medical issues [3].

Over time, physician–patient relationships have changed. Criticism of this paternalist approach to medical care, along with legislations protecting patients’ rights, led to a conceptual shift and the formation of a new approach: patient-centered care. This approach places patients and their problems and desires at the core of the encounter [4].

Research has shown that a good rapport is important in order to engage patients in follow-up treatment, especially for patients with chronic conditions, and that the encounter itself can have therapeutic effects [5,6,7,8,9]. Parallel to the changes in physician–patient relationships, researchers began to explore questions regarding the definition and traits associated with a “good” or “ideal” doctor. These traits can be roughly divided into those related to technical, professional skills and those related to interpersonal skills [10,11,12,13]. All physicians should apply relevant technical knowledge and skills in their practice. The question then arises as to whether a physician with strong technical skills but lacking in humaneness and interpersonal skills can be a “good doctor”.

Effective communication plays a key role in developing the physician–patient relationship and developing trust [14,15]. Patients who perceive their physician as caring and sensitive to their needs express greater satisfaction with their health care [16,17]. Further, physicians’ attention to patients’ emotional needs has a positive effect on recovery and responsiveness to treatment [18,19]. A study conducted in a mental health hospital in Israel found that patients want doctors and nurses to respect them as human beings and not to see them only as “cases”, to include them in decision-making, and to provide emotional support [20]. A study at Kaplan Medical Center in Israel surveyed 445 hospitalized patients about the characteristics of a “good doctor” [21]. The researchers compiled a questionnaire including 21 items covering three areas of medical care: patient autonomy, physician professionalism, and humaneness. The study finds that patients’ priority is for doctors to respect their autonomy, followed by wanting their doctor to be professional and humane. Similarly, a survey of 289 clients of 10 pharmacies in New Zealand finds that, from a given list of traits, the highest importance was attributed to patient autonomy and concern for patients’ well-being [21]. A review of 19 articles on patient perceptions of a “good doctor” in the context of primary health care finds that the most important aspect proved to be “humaneness’”: this was ranked highest in 86% of the studies that included this aspect. The second most important aspect was “competence/accuracy’” (64%), while “patients’ involvement in decisions” was the third most important aspect (63%) [13].

In other studies, researchers indicate that in the absence of concrete information regarding physicians’ technical capabilities, patients assume they are receiving high-level medical care when a physician has strong interpersonal skills [22,23,24].

However, a study of 1193 patients from six primary care clinics in the United Kingdom finds that patients are more concerned that their physicians have strong technical skills [25]. From a given list of traits, patients ranked as most important the physicians’ ability to conduct a thorough physical examination and continuity of treatment (so the physician is familiar with the patient).

In summary, most studies indicate that for public views humaneness, respect for patient autonomy, and cooperative decision-making as crucial traits for physicians [20,21,22,23,24]. As doctor–patient relationship is an important component of medical care [14], it is important to understand whether a similar or different picture emerges among physicians.

In the early 20th century, in response to growing emphasis on clinical competence and the scientific basis of the profession, various voices in the medical profession reminded colleagues of the need for humanness in addition to technical skill [26]. Claims were made that physicians must possess the virtues of humility, honesty, morality, integrity, and compassion, and must be devoid of any interest in personal gain, and that deviating from these values harms the “soul” of the medical profession [26,27,28].

These ideas have been noted in previous studies conducted among physicians, such as one done at Soroka University Medical Center in Israel [10]. When asked to rate the most important trait of a “good doctor” from among a list of six possibilities, physicians ranked the traits in the following order (from most to least important): humane and considerate treatment of patients, medical knowledge and skills, dedication and willingness to help patients, good relationships with staff, research and publishing ability, management and administrative skills. It is striking that, while empathic behavior is considered the most important attribute for being a “good doctor”, it was ranked last in the scale of importance for being promoted at the hospital. The researchers concluded that one physician may invest in relationships with patients in order to receive appreciation, while another may put more effort into doing research in order to gain scientific recognition and advance up the hospital hierarchy.

Similar findings emerge from a study conducted at the Tel Aviv University School of Medicine, in which 174 faculty members and 214 students in their preclinical and clinical years were surveyed [29]. They rated the five most important attributes as: honesty, a humane approach to patients, responsibility, professional skills and knowledge, and the ability to discriminate between important and minor issues. These researchers assert that socialization towards the image of the ideal physician, as indicated by these findings, is not implemented in medical school curricula, leading to a curricular reform.

Research conducted in the Netherlands indicates that physicians and patients do not always share the same priorities [30]. Surveyed patients attributed significantly higher priority to adequate consultation time, physician availability, and receiving detailed information about their condition, while the physicians emphasized care coordination and continuity. The researchers view these differences as an example of paternalism in medical care. Patients want to be informed and empowered consumers, while physicians prefer a long-term relationship with an obedient patient.

Previous research examines perceptions among physicians only or among patients only, but very few studies compare the perceptions of these two populations [30,31]. In addition, in most of the previous studies, participants were given an existing set of features to rate, whereas in the present study, participants were asked an open-ended question that allowed more complete information on the issue. Moreover, most of the research done in this context is over two decades old. Since then, there have been significant changes in the medical profession, the work environment, medical education, and medical technology [4]. Most notably, computerization in medical care has greatly influenced physician–patient relationships. Therefore, there is a need to reexamine current perceptions about the characteristics of a “good doctor”. It is clear that a “good doctor” cannot be defined solely by medical professionals but must also be derived from patients’ experiences in healthcare encounters with physicians. A profile of the traits considered most important for physicians can serve as a basis for accepting, training, and evaluating medical students, and for selecting educators to serve as roles model for future physicians.

The aims of the current study are to compare the perceptions of the general public with those of physicians from different specialties, including family medicine, regarding the most important features of a “good doctor” and regarding whether it is the physicians’ role to reduce health disparities. We hypothesize that the general public is more likely to define a “good doctor” as having interpersonal skills and traits of humaneness traits as compared to physicians who attribute more emphasis to technical and professional skills.

## 2. Materials and Methods

### 2.1. Study Design, Participants and Procedure

The study was designed as a cross-sectional study, conducted via two telephone surveys.

Physicians: A total of 2300 physicians were contacted by telephone and 1000 (43%) completed the questionnaire. The survey organization that completed the fieldwork (Dialogue—Organizational Consulting, Research and Training Ltd.) used a sample of specialist-physicians registered with the Israeli Medical Association (IMA), stratified according to their medical school training, which was found to affect physicians’ attitudes about their roles in a prior study.12 The survey was conducted in August 2016.

The General Public: A total of 500 members of the public were contacted and deemed eligible for a national telephone survey, constituting a representative sample of the adult population in Israel, and stratified by gender, age group, and residence region. Respondents were recruited until a representative stratified sample of 500 respondents was reached. The response rate was 27%. The telephone interviews were conducted in Hebrew, Russian or Arabic, according to the language of the interviewee, and lasted several minutes on average (3–10 min range). Three attempts were made before moving to the next interviewee. Respondents were not given a financial incentive to participate. The margin of error was ±5%.

### 2.2. The Survey Questionnaire

The research tool is a questionnaire consisting mainly of multiple-choice questions written by the researchers (see Appendix A). A pilot survey was conducted among 10 individuals from the adult population in the State of Israel, and 10 specialist-physicians. The general public and physician were asked about their socio-demographic characteristics and what they perceive as the first and second most important traits of a physician. In order to avoid bias, participants were asked in open-ended questionnaire items to name the traits they think are appropriate, and not to choose from a list of given traits. In addition, respondents were asked to what extent they think it is physicians’ role to reduce health disparities (along a 7-point scale, with 1 = not at all and 7 = to a large extent). This variable is differentiated into three categories: to a small extent (answers of 1–3), to a medium extent (answer of 4) and to a great extent (answers of 5–7). This question was drawn from an online survey of physicians [32]. It reflects a broad general view regarding whether physicians have a social and political role, beyond their obvious role as caregivers.

### 2.3. Data Analysis

The physician traits were coded into the two categories (technical and professional skills vs. non-technical interpersonal skills and humaneness) by researchers (KD, ND, and YGB) using a grounded theory approach. Grounded theory sets out to discover or construct theory from data, systematically obtained and analyzed using comparative analysis. The emerging codes are circulated among researchers and the list of codes is sorted in a face-to-face meeting. Once the researchers agree on the developed codes (to ensure fidelity) the data are further analyzed until conceptual saturation is reached, that is, no new codes or categories are generated. After this phase, the list of attributes was distributed to 16 experts from medicine, nursing, psychology, and sociology, who were asked to indicate whether they thought each attribute belonged to the category of technical/professional skills or non-technical interpersonal and humaneness skills. When there was disagreement, the data were further discussed in conference calls until complete consensus was reached.

### 2.4. Statistical Analysis

We compared the survey responses by testing differences between physicians and the general public using a chi-squared test (*χ*^2^) using SPSS v25 software (IBM, Armonk, NY, USA). This considered the design effects for each of the surveys by calculating the effective sample size. To adjust for sampling biases due to the sociodemographic differences in nonresponse rates and to ensure that the sample was representative, we compared and found no significant differences between respondents and nonrespondents for sex, age, level of education, and years of experience. All reported P values are based on 2-sided tests and were considered significant when below 0.05.

## 3. Results

### 3.1. Respondent Demographics

The Respondents’ characteristics are shown in Table 1 and Table 2. There were significant demographic differences between the samples of the physicians and the general public. The general public was more equal in terms of gender (with slightly more females), younger, had a lower level of education, and overall, had a lower reported financial status as compared with the physician population.

### 3.2. Perceptions of Physicians’ Role in Reducing Health Disparities

The question about physicians’ role in reducing health disparities used a seven-point Likert scale (1 = not at all and 7 = to a large extent). We categorized the results into three groupings: (1) to a small extent (answers of 1 to 3); (2) to a medium extent (answer of 4); and (3) to a great extent (answers of 5 to 7). There were significant differences between the physicians’ perceptions and those of the general public regarding the physicians’ role in reducing health disparities (*χ*^2^ = 13.40; *p* < 0.001). Among the members of the public, 41% (*n* = 205) said that reducing health disparities was a physicians’ role to a small extent, 48% (*n* = 240) said that it was their role to a larger extent, and 11% chose the middle category. In contrast, about one-third of physicians (31%; *n* = 310) said it was their role to a small extent, 56% (*n* = 560) said it was their role to a large extent, and 13% (*n* = 130) chose the middle category.

### 3.3. Perceptions of the Most Important Features of a “Good Doctor”

After three iterations among an expert panel, an aggregation of attributes for both professional and technical skills categories was achieved. The resulting nine attributes in the humaneness category and even attributes in the professional and technical skills category are shown in Table 3.

Views of physicians: The most important attributes of a “good doctor” according to the physicians we surveyed was their humaneness, empathy, knowledge and professionalism, credibility and honesty, and caring and devotion, for a total of 74% (Table 3). The second most important attribute indicated by physicians was knowledge and professionalism, empathy, humaneness, credibility and honesty, and caring and dedication, for a total of 71%. The differentiation of the traits into the two broad commonly used categories (technical and professional skills versus non-technical, interpersonal skills and humaneness) revealed that 62% (*n* = 620) of physicians indicated attributes of humaneness as the most important trait, whereas 38% indicated professional and technical skills. The reverse picture is seen regarding their perception of the second most important attribute: 61% (*n* = 610) of physicians chose professional and technical proficiency, whereas 39% (*n* = 390) chose humaneness.

Combining the results of these two questions indicates that 46% (*n* = 460) of physicians put humaneness in first place and professional skill in second place; 23% (*n* = 230) of physicians put professional skills in first place and humaneness in second; 16% (*n* = 160) chose an attribute of humaneness; and 15% (*n* = 150) chose 2 professional skills.

A comparison by type of specialization found that more physicians specializing in internal medicine indicated two traits of humaneness, as compared to physicians in surgical specializations (56% vs. 43%, respectively; *χ*^2^ = 4.01; *p* = 0.045). The same pattern was seen among physicians who primarily work in community clinics versus in hospitals (68% vs. 42%, respectively; *χ*^2^ = 16.14; *p* < 0.001) and among physicians who do not have a managerial role as compared to physicians with a managerial role (55% vs. 40%, respectively; *χ*^2^ = 4.39; *p* = 0.036); nonresearching physicians versus research physicians (58% vs. 46%, respectively; *χ*^2^ = 4.22; *p* = 0.040); and younger physicians (1–10 years since completing internship) versus more senior physicians (11 years or more since completing internship) (64% vs. 54%, respectively; *χ*^2^ = 8.59; *p* = 0.004). There were no significant differences by respondents’ gender in the choice of attributes of a “good doctor”.

Views of the public: Members of the general public reported that the most important attributes of a “good doctor” are knowledge and professionalism, credibility and honesty, humaneness, listening and patience, for a total of 81%. They reported that the second most important attributes include knowledge and professionalism, humaneness, credibility and honesty, empathy and listening, for a total of 72%. The distribution of these attributes into the 2 general categories described above showed that 55% (*n* = 275) of the public chose professional and technical skills as the most important attributes and 45% (*n* = 225) chose humaneness. The same is seen for the second most important attribute: 53% (*n* = 265) chose professional and technical skills, whereas 47% (*n* = 235) chose humaneness.

We found that by combining the responses to the two questions, 32% (*n* = 160) of the surveyed public selected a professional attribute as the most important and humaneness as the second most important; 28% (*n* = 140) indicated humaneness as the most important and a professional skill as the second most important; and 24% (*n* = 120) cited two professional skills and 16% (*n* = 80) cited two aspects of humaneness.

We found significant differences in the combined variable among members of the public with different education levels (*χ*^2^ = 7.91; *p* = 0.015). Respondents with a high school education were more likely to cite two traits of humaneness (*n* = 265, 53%) as compared with those who had a vocational secondary education (*n* = 180, 36%) and those with an academic education (*n* = 155, 31%). There were no significant differences when stratified by gender, level of religiosity, or income levels regarding their views on the attributes of a “good doctor”.

### 3.4. Comparison of Perceptions Held by Physicians and the General Public

We found significant differences between the views of physicians and members of the public in regard to the most important attributes of a “good doctor” (*χ*^2^ = 36.46; *p* < 0.001). Members of the public are more likely to focus on professional skills (*n* = 275, 55%) than physicians (*n* = 390, 39%). There were also significant differences between the two groups in their choice of the second most important trait (*χ*^2^ = 8.83; *p* = 0.003), which trended in the opposite direction: physicians were more likely to select professional skills (*n* = 610, 61%) as compared with members of the public (*n* = 265, 53%).

Combining the results of the two questions reveals significant differences between the groups (*χ*^2^ = 44.97; *p* < 0.001). The same percentage of physicians and members of the general public (16%) cite two traits of humaneness; 15% of physicians versus 24% of the general public cite two professional skills; 46% of physicians versus 28% of the general public cite a trait of humaneness as the most important and professional skills as the second most important trait; and 23% of physicians versus 32% of the general public cite professional skills as the most important and humaneness as the second most important trait.

## 4. Discussion

The physician–patient encounter, a keystone of health care, is based on communication [7]. This has become especially true in the past few decades, following the development of patient-centered care [4]. Thus, understanding physicians’ and patients’ expectations is crucial. This study is distinctive in its comparison of physicians’ and patients’ perceptions of the most important qualities of a “good doctor”.

This discussion first addresses the physicians’ perceptions, then those of the general public, and finally a comparison of the two.

Physicians: The surveyed physicians assert that people in their profession must first and foremost possess virtues of humaneness, in addition to knowledge and professional skills. This finding is consistent with previous studies [10,26,29,33] and with the ethical code of the medical profession as stated by the American Medical Association [34]. When the physicians describe a holistic set of traits, rather than a single trait, the majority (69%) includes both traits of humaneness and professional skills. Only 16% cite two traits of humaneness and 15% cite two traits related to professional and technical skills.

The current study is comparable to previous research that included also medical students [29], in which physicians specializing in the field of internal medicine are found to be more likely to indicate traits of humaneness in the five most important traits of the “ideal physician” as compared to surgical specialists. It seems that the physicians who have more personal and long-lasting contact with patients (especially those working in primary care in the Israeli context such as family physicians, and internal medicine specialists) place greater importance on humaneness over technical skills. In maintaining lasting connections with their patients, as in Israel patients have the right to choose their own primary care physician, it is essential for these physicians to have the ability to maintain empathic and effective communication with them. The research findings are also upheld by a previous study that found that physicians classified as being compassionate and empathic tend to be significantly younger than physicians not described as having these traits [10].

The general public: The current study’s findings among the general public sample are inconsistent with the findings of the literature review. Previous research found that patients are more likely to emphasize traits of humanness as their first priority [13,20,21,22,24,35,36] contrary to the findings of the present study.

However, our findings are consistent with an analysis of 3000 reviews written by patients on a German site for physician ratings, which finds that patients’ most common concern (63%) had to do with assessing physician professional competence [37]. It has been said patients in the 21st century have come a long way, from the earlier view that they should “trust your doctor to know what is in your best interest,” to a view that “the doctor’s job is to bring you the best science and technical skill and you will decide what is in your best interest” [38] (p. 216). That is, in an age in which patients have become consumers, the role of the physician is to be scientifically and technically proficient. It is possible that the public’s perceptions found in the present study reflect these changes, as well as deep concerns regarding the changing goals of the medical profession. In the past, commitment to patient well-being was the basis of the relationship. Today the rules of the game have changed to market-oriented health care with a service provider-consumer relationship [32]. Nevertheless, we should remember that sometime competition can force service providers to deliver services that are more patient-centered and technically competent since consumers may not choose them. Moreover, as part of the shift from a paternalistic model to a more equitable collaborative encounter, there are now physician rating websites (PRW). These move the physician–patient relationship to the next level: the patient becomes a consumer with the power to assess and publicly evaluate the health services received from physicians [31]. A survey of 1505 participants found that about a quarter of them had used such sites when searching for a physician, and 11% had published a rating on a PRW [39].

Level of education has a significant impact, as those with a high school education are more likely to indicate traits of humaneness in relation to those with post-secondary professional or academic education. This confirms the findings of a previous study, which also finds that, when describing the traits of a “good doctor”, patients without a college or university education gave higher ratings to interpersonal traits (such as: empathy, cooperative decision-making, friendliness of physician and staff, and patient satisfaction with treatment) as compared to patients with higher academic education [31]. The latter may have a greater ability to seek medical knowledge for themselves and to understand the importance of medical treatment for their health, so they have a greater need and expectations for their physician to strengthen their confidence by demonstrating knowledge and professionalism, rather than by being merely empathic. Various studies [31,40,41] have identified factors such as socioeconomic status, age and gender, that increase the need for engagement and sharing, but no such trends are found in the present study. These differences may be since most of the participants in the current study were relatively young and healthy, while much of the previous research was conducted among populations of patients in a vulnerable physical and/or emotional state, who therefore expressed greater need for humaneness from their physicians. Additionally, the differences in findings may be due to the use of different methods for measuring the variables. Previous studies gave patients a list of traits to rate, while in the present study participants were asked to answer the question in an open and intuitive way.

### 4.1. Comparison of Physicians and the General Public

The findings show that, contrary to the research hypothesis, physicians place more emphasis on humaneness while the general public places greater emphasis on professional and technical skills. In addition, it is found that the general public is less likely to say that working towards reducing health disparities is the task of physicians; the physicians themselves are more likely to see this as part of their role. This difference in perception may be because patients perceive the physicians’ role as helping them return to optimal functioning [42] and are less likely to see the social and political significance inherent in the physician role. In contrast, physicians may assume that specialist-physicians have already acquired a high level of technical skill, and therefore emphasis should be placed on the human and social-political aspects of their work [32]. This finding correlates with the fact that often the public (including in Israel) pushes for the “medicalization” of illness, including demands and social protest for enlarging publicly funded health care, rather than demanding the improvement of social determinants of health [9].

The differences between physicians’ and patients’ perceptions found in this study are consistent with those of a previous study [31] which finds that physicians rated interpersonal capabilities as more important than technical skills, whereas the public assesses technical skills as more important than interpersonal skills when describing a “good doctor”. Paterson [28] agrees with this argument and writes that, as a general rule, patients can judge physicians’ personality traits, but cannot assess their clinical skill, and therefore should trust them on the latter. Patients want to assume that their physicians are adept at doing their job and at recognizing the limits of their professional abilities [22,23,24]. It may be that the participants in the current research prefer a high level of professionalism in a “good doctor” because they are better able to judge interpersonal skills on their own, and thus can choose a physician with whom they personally connect. In addition, patients may understand why physicians are often emotionally detached, and some even prefer to keep their contact on a professional basis. Moore [43] adds that medical care is influenced by politics and that it is difficult to uphold a humanistic view of medicine when the rules are adapted for efficient management and the imperative to make a profit. It is possible that the public is aware of this spirit of the modern age and therefore has fewer expectations about humaneness and is content with professional skills.

Deborah Lupton [44] argues that in the medical encounter, physicians and patients work together to produce results that benefit them both. To improve the patient’s health, they must cope together with the uncertainty that characterizes any medical encounter [45]. The uncertainty experienced by both patient and physician partly shapes their interaction, and this cannot be reduced to a simple explanation in terms of domination or exploitation. Thus, patients might be more interested in what they perceive will most benefit them (the medical component). In contrast, even physicians who feel they possess accurate scientific knowledge are aware of the uncertainties inherent in the medical professions, perhaps even more so than the patient. This awareness shapes the interaction, demanding collaboration between doctor and patient. Thus, physicians emphasize the aspect of humaneness, in order to improve the interaction.

Compared to the public, physicians seem to be clinging to the ideal of healing and ethical relationships, in which they are ideally able to put themselves in the patients’ place and give them a sense of camaraderie and identification. Moreover, beyond the professional perspective, many physicians also have insights from their own occasional experiences of needing medical treatment.

### 4.2. Study Limitations

As with any survey data being self-reported and not aligned with objective measures, there is always the possibility of information bias and possibly some social desirability bias, mainly on the part of the physicians. The main limitation of the current research concerns the dichotomous definitions and distribution of traits of humaneness versus professional skills. This division that was classified dichotomously can be a basis for a future discussion. It is possible that some of the respondents interpreted professionalism as a purely technical aspect, whereas others may interpret professionalism according to behavioral traits of a “good doctor” (integrity, compassion, altruism, and ethics). The classification of traits into these content categories depends on personal views as well as professional, social, and cultural norms. This is reflected in physician’s distribution of the list of traits, which made it evident that some physicians believe that almost all the traits belong to the world of professional skills, while others believe they can be classified dichotomously, as described in the article.

As response rate was lower for patients (27%) than among physicians (43%), we should be cautious when extrapolating and generalizing these results, especially as non-response bias analysis was not available. In addition, as this study was part of a larger research project only recently completed, survey answers were given several years ago. In an era of fast transformations in medical care, it means that we should be cautious when generalizing from this study to our current healthcare system.

Another limitation concerns the representativeness of the sample of physicians in Israel. Due to the nature of the broader funded research project, the survey was conducted only among graduates of medical schools in Israel, although many physicians working in Israel completed their studies abroad. As these physicians are less represented in the Israeli Medical Association where we conducted our survey and as this study was part of a larger project related to the need for medical education reform in Israel, we decided to focus on the graduates from Israeli medical schools and further research is needed. Moreover, there may be intercultural differences that have not been examined in the present study.

## 5. Conclusions

While the medical profession is one of the most technologically advanced professions, one of the dangers inherent in innovation and technological advancement is that these will come at the expense of physicians’ emotional connection with their patients [46]. Patients today must rely on physicians’ scientific, clinical, and ethical abilities. Physicians’ commitment to the welfare of the patient serves as the basis for creating trust between them. Physicians need to put the best interests of patients first, within the political, economic, and consumer-oriented climate that characterizes the 21st century. Accelerated technological development and use of medical science for purposes quite different from its historical goals of preventing suffering and promoting health, alongside the medicalization of many aspects of modern life, make it necessary for there to be a public discussion of the moral core and changing goals of the medical profession.

The profile traits that emerged in this study can serve as a basis for accepting and evaluating medical students, training these students, and selecting educators to serve as role models for the humaneness of the medical profession among future physicians. The two aspects of health care—professional and technical skills versus humaneness and interpersonal skills—represent different skill sets. The former is based on technical knowledge and skills, the second is based on emotional warmth and communication skills. The ability to connect with patients and gain their trust is a human capability which crosses the boundaries of medical skills and knowledge. Therefore, it is important that the training and education of future physicians also focus on embedding this aspect in their work. As Morgan Martin [47] (p. 2776) writes: “Physicians dispense not only medicines but words that influence medicines or, all by themselves, affect the patient more than the medicine”.

Future research should include additional methodologies, such as combined focus groups of physicians and public representatives. It could also consider ethical dilemmas regarding the medical encounter. Such encounters could provide insights for physicians and the public about their expectations of one another.

## Figures and Tables

**Table 1:** Physicians’ Characteristics.

Character	%/Avg.
Men (%)	70
Age (Average ± SD) Range: 33–66 years	47 ± 6.58
Years of seniority as a specialist (Average ± SD) Range: 1–33 years	10 ± 6.33
Israeli born (%)	81
Specialty (%)	
Primary care specialties (family and internal medicine)	66
Surgical specialists	30
Diagnostic fields	4
Main Workplace (%)	
Hospital	63
Community	31
Research or Management	6
Managerial role (%)	23
Engaged in research (%)	56

**Table 2:** Public Respondents’ Characteristics.

Character	%
Men (%)	47
Age Group (%)	
18–34	33
35–44	17
45–54	16
55–64	16
65+	18
Married (%)	67
Israeli born (%)	69
Level of Education (%)	
high school education	40
vocational secondary education	20
Academic Education	40
Socio-Economic Status (%)	
Below Average	39
Average	35
Above Average	26
Religiosity (%)	
Secular	42
Traditional religious	30
Orthodox religious	16
Ultra-Orthodox	9
Did not respond	3

**Table 3:** Distribution of Views on Key Traits of a “Good Doctor”.

List of Triats	Trait Categorization as Humanness (H) or Professional (P)	Trait Noted as First Most Important (%)		Trait Noted as Second Most Important (%)	
		Physicians (*n* = 1000)	Public (*n* = 500)	Physicians (*n* = 1000)	Public (*n* = 500)
Humaneness/humane approach	H	20	16	9	16
Empathy	H	17	5	11	7
Caring and devotion	H	8	5	6	4
Patience	H	4	7	3	4
Attentiveness	H	4	8	3	7
Love of humanity	H	3	-	2	-
Communicativeness	H	2	2	2	5
Humility	H	2	-	2	-
Courtesy	H	-	2	1	3
Professionally knowledgeable	P	15	33	36	32
Credibility and honesty	P	14	17	9	10
Diligence and perseverance	P	3	-	4	-
Curiosity	P	2	-	4	-
Responsibility	P	1	-	1	1
Love of the profession	P	1	-	-	-
Accuracy in diagnosis	P	1	3	2	6
No answer	-	3	2	5	5

## Data Availability

The data that support the findings of this study are available from the corresponding author.

## Abbreviations

AMA: American Medical Association; IMA: Israeli Medical Association; PRW: Physician Rating Websites.

## Appendix A. Questionnaire for Physicians

1. Year of graduation in medical school? _________
2. Year of completion of the internship? _________
3. What is your area of expertise? ____________
5. What is your main workplace?
6. What is your role?
7. Do you have an academic appointment? 0. No 1. Yes
8. Are you involved in research? 0. No 1. Yes
9. What do you think are the two most important qualities in a doctor, with the first being the most important? __
10. What trait, in your opinion, can make a "doctor" a "good doctor"? ____
11. Do you work, or have you worked at a clinic located in a periphery area?
12. Do you think it is the role of the doctor to reduce health disparities between population groups? On a scale ranging from 1–7, when: 1- not at all; 7- To a very large extent
13. Sex: 0. Male 1. Female
14. Year of birth: _____
15. Where were you born: 1. Israel 2. Other: ___
16. Religion: 1. Jew 2. Muslim 3. Christian 4. Other: _____
